# A Keen Diagnosis

**Published:** 1899-10

**Authors:** 


### A Keen Diagnosis.

A somewhat distracted mother, who was also a little deaf, brought her daughter the other day to see a Birmingham physician. The girl was suffering from what is known among many people as "general lowness." There was nothing much the matter with her, but she was pale and listless, and did not care about eating or doing anything. The doctor, after due consultation, prescribed for her a glass of claret three times a day with her meals. In ten days' time they were back again, and the girl looked quite a different creature. She was rosy-cheeked, smiling and the picture of health. The doctor congratulated himself upon the keen insight he had displayed in the diagnosis of the case. "Yes," exclaimed the excited and grateful mother, "thanks to you, Doctor. She has had just what you ordered. She has eaten carrots three times a day since we were here, and sometimes oftener—and once or twice uncooked—now look at her!" —*Med. Times.*

